# Concomitant decalcification of the anterior mitral leaflet via the aortic annulus during aortic valve replacement for significant aortic and mitral stenoses

**DOI:** 10.1002/ccr3.5119

**Published:** 2021-11-25

**Authors:** Akihisa Furuta, Shogo Mukai, Hironobu Morimoto, Junya Kitaura, Daisuke Futagami

**Affiliations:** ^1^ Department of Cardiovascular Surgery Fukuyama Cardiovascular Hospital Fukuyama Japan

**Keywords:** anterior mitral leaflet, concomitant decalcification, mitral calcification

## Abstract

An 82‐year‐old man undergoing regular hemodialysis with substantial aortic and mitral valve stenoses underwent aortic valve replacement with concomitant mitral decalcification via the aortic annulus. Postoperative transthoracic echocardiography showed reduced mitral stenosis. The patient was discharged on the 14th postoperative day uneventfully.

## Graphical abstract

Concomitant decalcification of the anterior mitral leaflet via the aortic annulus during aortic valve replacement may be effective in reducing mitral stenosis in high‐risk patients with severe aortic and mitral valve stenoses within short aortic cross‐clamp time.

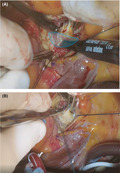

## 1 INTRODUCTION

Significant MS caused by mitral calcification is sometimes accompanied by aortic stenosis (AS).^1^, ^2^ In high‐risk patients with both substantial AS and MS, double valve replacement can increase the risk of mortality and critical complications, and less invasive alternatives should be performed.

Herein, we report a case of concomitant decalcification of the anterior mitral leaflet during aortic valve replacement, which effectively reduced MS through a minimally invasive approach.

## 2 CASE REPORT

**FIGURE 1:**
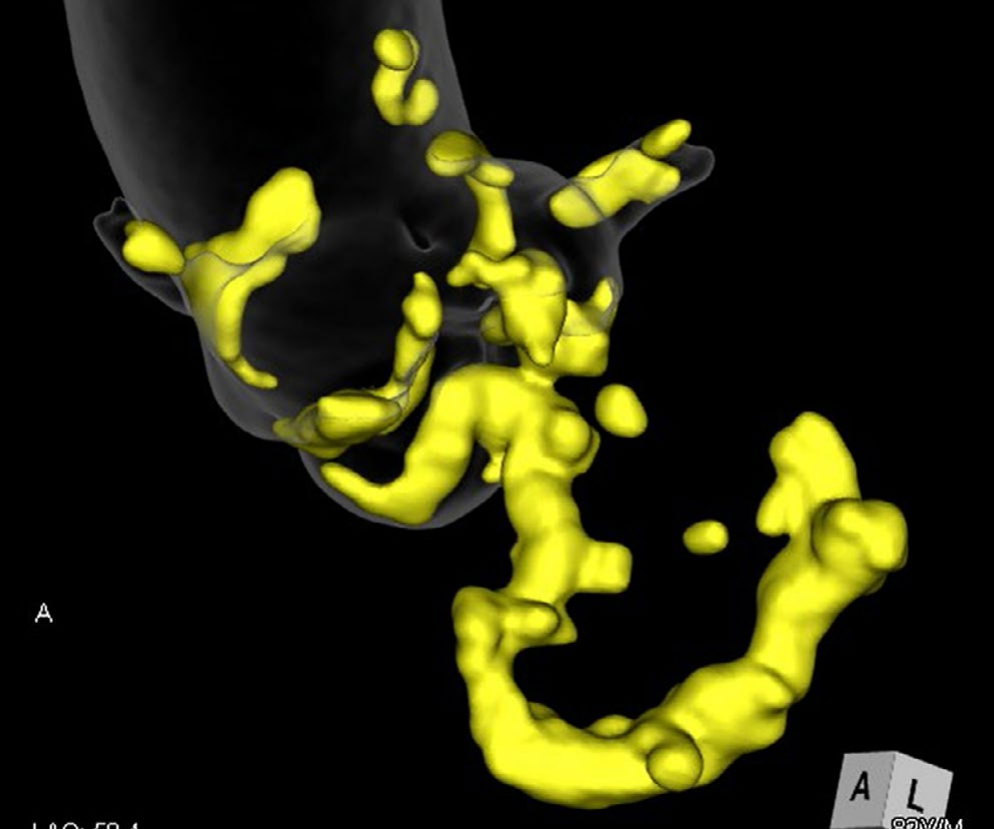
Preoperative three‐dimensional reconstructed computed tomography images. Severe calcifications ranged continuously from the noncoronary cusp to the left ventricular outflow tract and mitral annulus. Mitral annular calcification was observed in three‐fourths circumference (half of the anterior leaflet and entire posterior leaflet)

An 82‐year‐old man with a history of regular hemodialysis for chronic renal failure presented with dyspnea. The patient had no history of rheumatic fever. Transthoracic echocardiography indicated significant AS (transaortic peak velocity, 4.8 m/s; planimetered valve area, 0.64 cm^2^) and MS (transmitral peak velocity, 2.2 m/s; mean gradient, 7 mmHg; pressure half time, 152 ms; mitral valve area, 1.45 cm^2^) with restricted motion of both the anterior and posterior mitral leaflets due to calcification without commissure fusion, mild aortic and mitral regurgitation, and normal left ventricular function (ejection fraction, 63%). Three‐dimensional computed tomography images revealed continuous severe calcification ranging from the noncoronary cusp to the mitral annulus via the left ventricular outflow tract (Figure 1).

The predicted operative mortality rates derived from the EuroSCORE II in the case of isolated aortic valve replacement and double valve replacement were 4.74% and 7.92%, respectively. Considering the high mortality and morbidity rates, we selected to perform concomitant decalcification of the anterior mitral leaflet during aortic valve replacement over double valve replacement.

**FIGURE 2:**
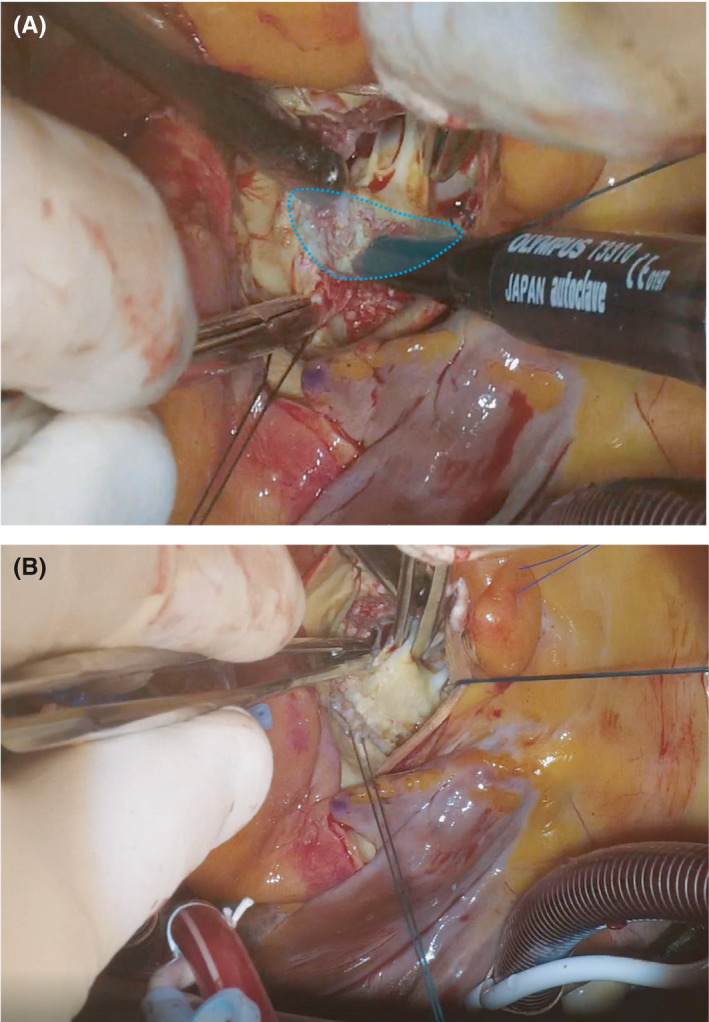
Operative findings. (A) The calcification of the anterior mitral leaflet at the left ventricular side was removed using an ultrasonic surgical aspirator. The blue dotted line indicates the decalcification range. There was no subvalvular fusion or shortening of the chordae tendineae. (B) The surface of the anterior mitral leaflet was smoothened after decalcification of the left ventricular outflow tract and mitral anterior leaflet

**FIGURE 3:**
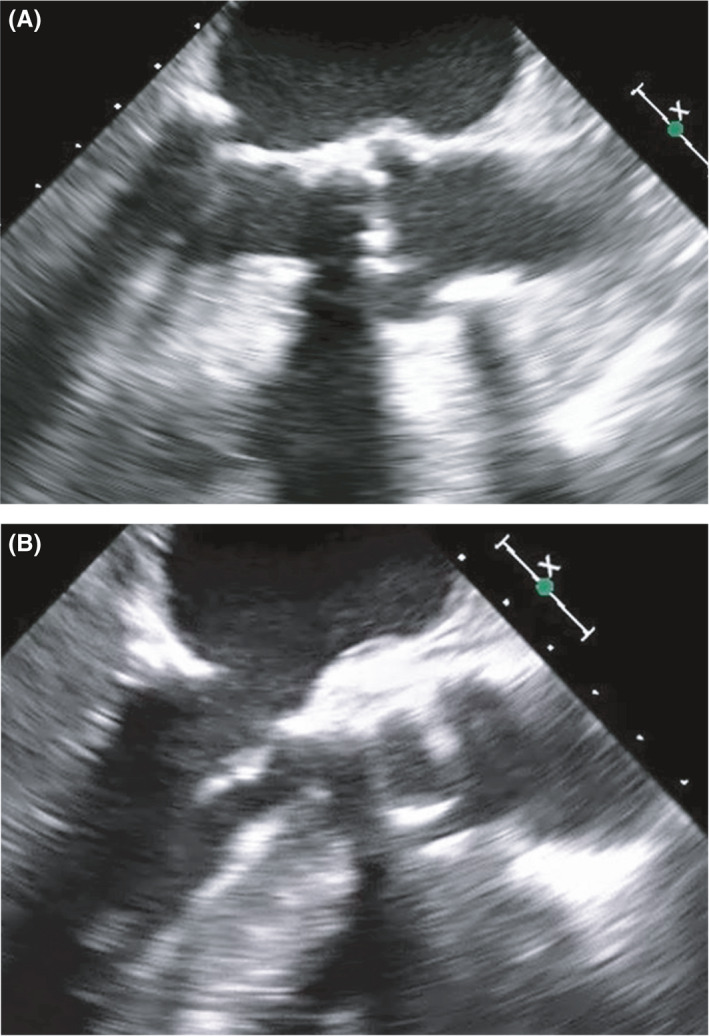
Pre‐ and post‐procedure transesophageal echocardiography in the diastole phase. (A) Pre‐procedure (125 degrees). (B) Post‐procedure (125 degrees). Compared with the pre‐procedure anterior mitral leaflet (A), the post‐procedure anterior mitral leaflet more extensively and smoothly opened in the diastole phase (B)

Under general anesthesia, cardiopulmonary bypass was established at systemic temperature via ascending aortic and bicaval cannulation after a median sternotomy. After resection and decalcification of the aortic valve, decalcification of the left ventricular outflow tract and the anterior mitral leaflet at the left ventricular side was performed using ultrasonic surgical aspirator (Sono Surg; Olympus, Tokyo, Japan) via the aortic annulus (Figure 2). Aortic valve replacement was completed using a bioprosthetic valve. Postoperative transesophageal echocardiography demonstrated a wider motion range of the anterior mitral leaflet than the preoperative range (Figure 3 and Video S1). The cardiopulmonary bypass and aortic cross‐clamp times were 136 and 100 min, respectively.

Postoperative transthoracic echocardiography showed mitral regurgitation remained less than mild. The transmitral peak velocity, mean gradient, and pressure half time decreased from 2.2 m/s to 1.6 m/s, from 7 mmHg to 4 mmHg, and from 152 ms to 130 ms, respectively, and the mitral valve area increased from 1.45 cm^2^ to 1.70 cm^2^. The patient was discharged on the 14th postoperative day uneventfully.

## 3 COMMENT

Our patient was diagnosed with significant AS and non‐rheumatic MS according to the preoperative echocardiography and patient's history. Because transcatheter aortic valve replacement in patients undergoing dialysis is not covered by insurance in Japan, we decided to perform surgical aortic valve replacement. Owing to the high predicted operative mortality rate and other critical complications such as major bleeding and left ventricular rupture in the case of double valve replacement, we decided to perform concomitant decalcification of the anterior mitral leaflet during aortic valve replacement.

Concomitant decalcification of the anterior mitral leaflet via the aortic annulus was conducted in a relatively wide visual field and reduced MS effectively within short aortic cross‐clamp time as shown in our case. However, this technique might be exclusively limited to some patients: MS caused by the anterior leaflet calcification with or without the posterior leaflet calcification is especially feasible, while MS related to subvalvular fusion, shortening of the chordae tendineae, and commissure calcification and MS accompanied by significant mitral regurgitation might not be suitable for this technique. To avoid inadequate decalcification causing MS to persist and excessive decalcification resulting in leaflet perforation, attention should be given to the color, configuration, and stiffness of the mitral leaflet.

The most serious concern about this procedure is long‐term durability. Because there is little literature concerning decalcification of mitral valve, the long‐term incidence of mitral valve‐related complications remains unknown. We are not dealing with aortic valve decalcification, but it may be worth mentioning that both restenosis and regurgitation were reported to be found several years after aortic valve decalcification.^3^ As these complications could occur after mitral decalcification, a long‐term follow‐up is indispensable.

## 4 CONCLUSION

Concomitant decalcification of the anterior mitral leaflet during aortic valve replacement was a minimally invasive approach and effective in reducing mitral stenosis in high‐risk patients with substantial AS and MS. However, it is unknown how long the effect of this procedure continues, and thus, cautious follow‐up should be employed.

## CONFLICTS OF INTERESTS

None.

## AUTHOR CONTRIBUTIONS

Akihisa Furuta involved in study concept/design, drafting article and critical revision, and approval of the article. Shogo Mukai involved in study concept/design and approval of the article. Hironobu Morimoto involved in study concept/design and critical revision. Junya Kitaura involved in critical revision and approval of the article. Daisuke Futagami involved data interpretation and approval of the article.

## ETHICAL APPROVAL

Approval of the International Review Board was not required at our institution because this study was a case report and informed consent was obtained from the patient for publication.

## CONSENT

Written informed consent was obtained from the patient to publish this report in accordance with journal's patient consent policy.

## PERMISSION TO REPRODUCE MATERIAL FROM OTHER SOURCES

None.

## CLINICAL TRIAL REGISTRATION

None.

## Supporting information

Video S1

## Data Availability

The data that support the findings of this study are available from the corresponding author upon reasonable request.
